# Supplementary figures and images for: 2-methylquinazoline derivative F7 as a potent and selective HDAC6 inhibitor protected against rhabdomyolysis-induced acute kidney injury
Source: PLoS One. 2019 Oct 22;14(10):e0224158. doi: 10.1371/journal.pone.0224158 (PMC6804997; doi:10.1371/journal.pone.0224158)

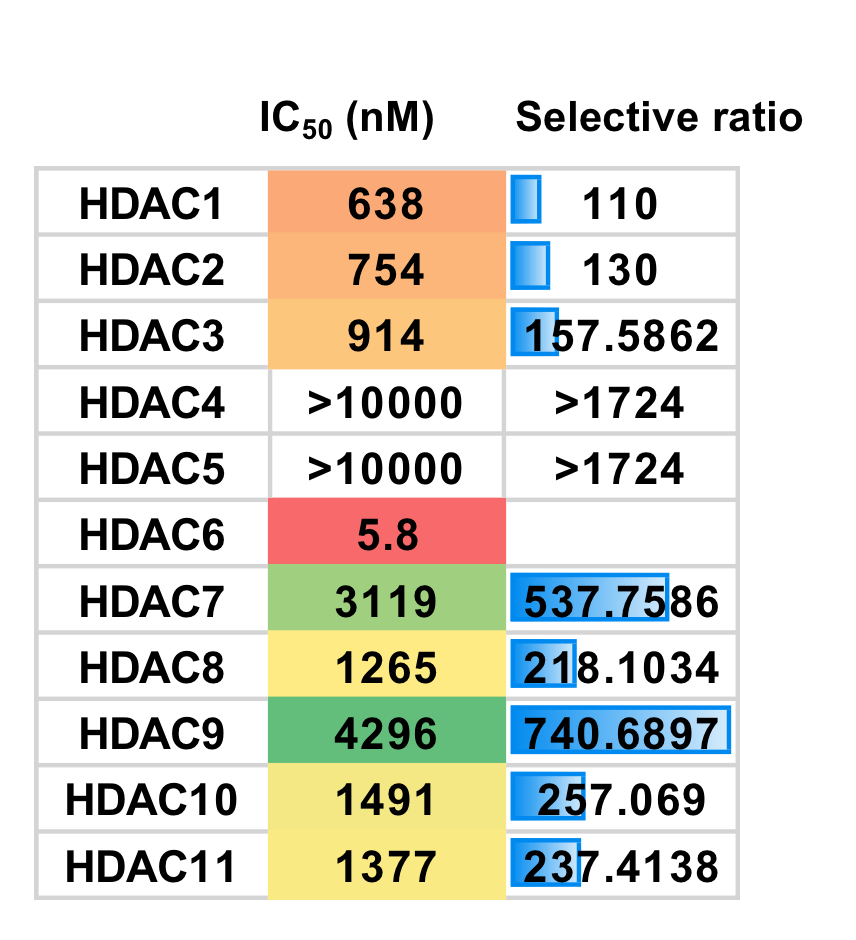

Supplement: S1 Fig — (TIFF) [file pone.0224158.s001.tiff]

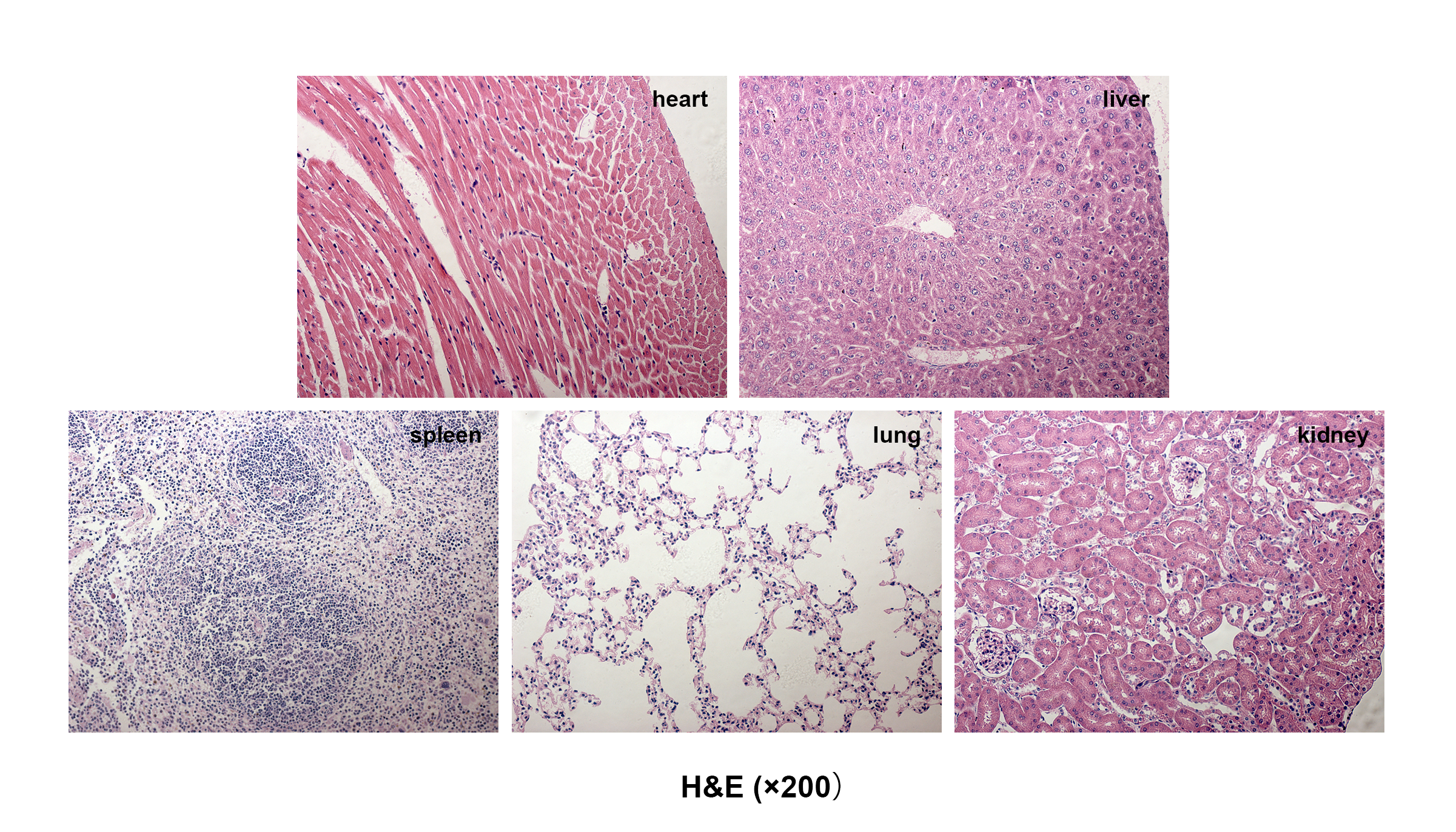

Supplement: S2 Fig — Photomicrographs (×200) illustrated H&E staining of the heart, liver, spleen, lung and kidney tissues from mice with oral administration of F7 at a dose of 40 mg/kg/d for 3 days but without glycerol injection. (TIF) [file pone.0224158.s002.tif]
