# Supplementary material for: 2-methylquinazoline derivative F7 as a potent and selective HDAC6 inhibitor protected against rhabdomyolysis-induced acute kidney injury
Source: PLoS One. 2019 Oct 22;14(10):e0224158. doi: 10.1371/journal.pone.0224158 (PMC6804997; doi:10.1371/journal.pone.0224158)
Supplement: S1 Table — (PDF) [file pone.0224158.s003.pdf]

S1 Table | Comparison of HDAC Inhibition Activity among HDAC6 inhibitors.

HDACs	F7		23BB		ACY-1215		LBH-589		SAHA	
	IC50 (nM)	Selective Ratio	IC50 (nM)	Selective Ratio	IC50 (nM)	Selective Ratio	IC50 (nM)	Selective Ratio	IC50 (nM)	Selective Ratio
**HDAC1**	638	110	422	24.824	38	4.22	1	0.25	11	0.73
**HDAC2**	754	130	386	22.706	95	10.56	3	0.75	35	2.33
**HDAC3**	914	157.59	439	25.824	135	15	2	0.5	30	2
**HDAC4**	>10000	>1724.14	>10000	>588.235	>10000	>1111.11	338	84.5	>10000	>666.67
**HDAC5**	>10000	>1724.14	>10000	>588.235	>10000	>1111.11	190	47.5	>10000	>666.67
**HDAC6**	5.8	-	17	-	9	-	4	-	15	-
**HDAC7**	3119	537.76	>10000	>588.235	>10000	>1111.11	4354	1088.5	>10000	>666.67
**HDAC8**	1265	218.10	3398	199.882	254	28.22	5	1.25	172	11.47
**HDAC9**	4296	740.69	>10000	>588.235	>10000	>1111.11	888	222	>10000	>666.67
**HDAC10**	1491	257.07	1176	69.176	194	21.56	4	1	170	11.33
**HDAC11**	1377	237.41	>10000	>588.235	>10000	>1111.11	4412	1103	>10000	>666.67
