# Supplementary material for: 2-methylquinazoline derivative F7 as a potent and selective HDAC6 inhibitor protected against rhabdomyolysis-induced acute kidney injury
Source: PLoS One. 2019 Oct 22;14(10):e0224158. doi: 10.1371/journal.pone.0224158 (PMC6804997; doi:10.1371/journal.pone.0224158)
Supplement: Attachment — Submitted filename: comment-2.docx [file pone.0224158.s004.docx]

In this study, Liu et al. have identified a novel HDAC6 inhibitor F7can alleviate rhabdomyolysis-induced AKI through inhibition of NF-kappB and ERK1/2 signaling pathway and reduction of IL-beta expression. This is an interesting observation and provides a novel inhibitor HDAC6. The manuscript may be improved through addressing following concerns.

1. In Figure 3D, expression of phospho-ERK1/2 and NGAL should be examined by immunoblot analysis.
2. In figure 3A, Glycerol was missed in the second picture
3. In Figure 4B, it seems that F7 treatment does not affect Mb-induced deacetylation of H3. The authors may examine whether F7 affect acetylation of alpha-tubulin in order to reflect its inhibitory effect in vitro.
4. The authors should discuss the comparison about the efficacy, chemical property or administration route etc between F7 and 23BB, another HDAC6 inhibitor synthesized by this group.
